# Risk and Prognostic Factors of Inpatient Mortality Associated with Unintentional Insecticide and Herbicide Poisonings: A Retrospective Cohort Study

**DOI:** 10.1371/journal.pone.0045627

**Published:** 2012-09-21

**Authors:** Wu-Chien Chien, Chi-Hsiang Chung, Jouni J. K. Jaakkola, Chi-Ming Chu, Senyeong Kao, Sui-Lung Su, Ching-Huang Lai

**Affiliations:** 1 Department of Public Health, National Defense Medical Center, No. 161, Section 6, Neihu District, Taipei City, Taiwan, Republic of China; 2 Graduate Institute of Life Sciences, National Defense Medical Center, No. 161, Section 6, Neihu District, Taipei City, Taiwan, Republic of China; 3 Center for Environmental and Respiratory Health Research, Institute of Health Sciences, University of Oulu, Oulu, Finland; University of Montreal, Canada

## Abstract

**Introduction:**

Pesticide poisoning is an important public health problem worldwide. The study aimed to determine the risk of all-cause and cause-specific inpatient mortality and to identify prognostic factors for inpatient mortality associated with unintentional insecticide and herbicide pesticide poisonings.

**Methods:**

We performed a retrospective cohort study of 3,986 inpatients recruited at hospitalization between 1999 and 2008 in Taiwan. We used the International Classification of Disease, 9th ed., Clinical Modification external causes of injury codes to classify poisoning agents into accidental poisoning by insecticides and herbicides. Comparisons in mortality rates were made between insecticide poisoning patients and herbicide poisoning patients by using the Cox proportional hazards models to estimate multivariable-adjusted hazard ratios (HRs) and their 95% confidence intervals (CIs).

**Results:**

There were 168 deaths during 21,583 person-days of follow-up evaluation (7.8 per 1,000 person-days). The major causes of mortality for insecticide poisonings were the toxic effect of organophosphate and coma, and the major causes of mortality for herbicide poisonings were the toxic effect of other pesticides and the toxic effect of organophosphate. The mortality for herbicide exposure was fourfold higher than that for insecticide exposure. The factors associated with inpatient mortality were herbicide poisonings (HR = 4.58, 95% CI 3.29 to 6.37) and receiving mechanical ventilation treatment (HR = 3.85, 95% CI 2.73 to 5.42).

**Conclusions:**

We demonstrated that herbicides stand out as the dominant agent for poisoning-related fatalities. The control of and limiting access to herbicide agents and developing appropriate therapeutic regimens, including emergency care, should be priorities.

## Introduction

Pesticide poisoning is a significant public health problem worldwide. With the development of modern agriculture, patients are increasingly exposed to new types of insecticides and herbicides. Currently, the most common type of pesticide in Taiwan is organophosphate pesticides (OP) [1], and glyphosphate and paraquat are the two types of herbicides that are used most frequently [2].

Pesticide exposure in Taiwan was recently evaluated using the nationwide registry maintained by the network of Taiwan's Poison Control Centers (PCC) [3]. Over the course of eight years (1985–1993), 23,436 telephone calls concerning human poisoning exposure were recorded. The most frequent cause of poisoning was pesticides (29.3%). Among all fatal cases, paraquat and organophosphate exposures were the two leading causes of death [4]. Another study reported that there were 4799 organophosphate pesticide (OP) exposures from July 1985 through December 2006 [1], [5]. The fatality rate was 12.7%. These studies were based on information collected in telephone interviews on the topic of poisoning exposures. The source population was poorly specified, and therefore, these figures could not be used to estimate the incidence of poisoning. Furthermore, previous studies were of limited sample size and were largely based on one or few health-care facilities.

The National Health Insurance (NHI) in Taiwan was launched on March 1, 1995. Since then, medical institutions have been required by law to make inpatient claims to the NHI Bureau. Given that Taiwan's coverage rate is over 99%, the NHI Research Database provides a representative and evidence-based source of data for studies in this field. Our understanding of the fatality rate of pesticide poisoning has improved [6]. However, patients experiencing pesticide poisoning may experience multiple severe episodes during the same hospitalization or after discharge. Data on the contribution of subsequent episodes to the disease burden are still limited because most past studies used overall fatality as the measured outcome. Furthermore, little information is available on the inpatient hospital stay of pesticide poisoning patients and on the survival rates of patients hospitalized for pesticide poisoning.

During the 10-year period from 1999 to 2008, unintentional pesticide poisonings accounted for 6,328 hospitalizations in Taiwan [6]. To address the extent and major agents of exposure of acute pesticide poisoning in Taiwan resulting in hospitalization, we obtained data from a nationwide hospitalization database to determine the rate of all-cause and cause-specific inpatient mortality and to identify prognostic factors of inpatient mortality among insecticide and herbicide pesticide poisonings.

## Methods

The hospitalization data were retrieved from the NHI Research Database (NHIRD). Categorization of the poisoning rates, the causes of hospitalization, and the cause of death after hospitalization occurring between 1999 and 2008 was carried out using the International Classification of Disease, 9^th^ ed., Clinical Modification (ICD-9-CM). This study was conducted using a data base without patient identifications, thereby conforming to the Declaration of Helsinki.

Patients were categorized into six age groups: infants and toddlers aged 0–4, children aged 5–14, young adults aged 15–24, mature adults aged 25–44, middle-aged adults aged 45–64 and elderly aged 65 or older. We defined unintentional poisoning by using the ICD-9-CM external causes of injury codes (E-Codes) to classify poisoning types into accidental poisoning by insecticides of organophosphate (E863.1) and accidental poisoning by herbicides (E863.5). Because the most common type of insecticide in Taiwan is organophosphate insecticide (OP) [1], we excluded patients who presented with poisoning with organochlorine compounds (E863.0) (n = 27) or carbamates (E863.2) (n = 97), poisoning by a mixture of insecticides (E863.3) (n = 170), or poisoning by other and unspecified insecticides (E863.4) (n = 1197), fungicides (E863.6) (n = 9), rodenticides (E863.7) (n = 238), fumigants (E863.8) and other and unspecified agricultural and horticultural chemicals and pharmaceutical preparations other than plant foods and fertilizers (E863.9) (n = 508).

The NHI database recorded the status of prognosis after each hospitalization. Information on death after hospitalization was obtained from the variable of Trans_code from Inpatient expenditures by admissions (DD) data file. The operational definition of death after hospitalization is that the patient dies during hospitalization or immediately after leaving a hospital in a critical condition voluntarily. All cause and cause-specific mortality of inpatients due to unintentional insecticide and herbicide poisonings were defined by using ICD-9-CM N-Code. Cause-specific mortality of inpatients due to unintentional insecticide and herbicide poisonings were further categorized as toxic effect of organophosphates (989.3)(n = 64), toxic effect of other pesticides, not elsewhere classified (989.4)(n = 93), anoxic brain damage (348.1)(n = 1), cardiac arrest (427.5) (n = 1), congestive heart failure (428.0)(n = 1), pneumonitis due to inhalation of food or vomiting (507.0)(n = 1), acute respiratory failure (518.81)(n = 1), alcoholic liver damage, unspecified (571.3)(n = 1), acute renal failure (584.9)(n = 1), coma (780.01)(n = 2), and toxic effect of unspecified lead compound (984.9)(n = 2). Overall mortality analyses used time-to-event methods and were based on 21,583 person-days of inpatient follow-up evaluation. The person-days of follow-up evaluation were calculated from the date of hospitalization to inpatient death or the last date of the inpatient stay. Because the duration of hospitalization varies, the person-time approach is a useful way of expressing mortality taking into account differences in the hospitalization. For patients with multiple hospitalizations, all hospitalization days were summed. Mortality rates, expressed in no of deaths per 1000 person-days, were calculated by dividing the number of deaths by corresponding person time, and survival curves based on person time were derived by the Kaplan-Meier method. The log-rank test was used to compare the survival curves.

Comparisons in mortality rates were made between insecticide poisoning patients and herbicide poisoning patients by using the Cox proportional hazards models to estimate multivariable-adjusted hazard ratios (HRs) and their 95% confidence intervals (CIs). The covariates used in the analyses included the following: type of pesticide poisoning (insecticides/herbicides), age (0–4, 5–14,15–24, 25–44, 45–64, 65 or older), sex (male/female), Charlson Comorbidity Index (CCI), living area (northern, central, southern, eastern, offshore), level of care (medical center, regional hospital, local hospital), mechanical ventilation treatment (yes/no). “Catastrophic illness” meant that patients had one or more following diseases or injuries: malignant neoplasm, congenital deficiency of clotting factors, hemolytic or aplastic anemia, renal disease with renal failure, systemic lupus erythematosus, systemic sclerosis, rheumatoid arthritis, polymyositis, dermatomyositis, vasculitis, pemphigus, Sjogern's syndrome, Crohn's syndrome, ulcerative colitis, schizophrenic disorders, disorders of metabolism, congenital anomalies, burn of >20% of total body surface, complications of transplant, acute poliomyelitis with paralysis, injury severity score ≧16, used respirator 6 hours per day continue 30 days, a fully intravenous diet for 30 days, decompression sickness, air embolism, myasthenia gravis, disorder of immune mechanism, fracture of vertebral column with spinal cord injury, occupational disease, cerebrovascular disease with acute stage, multiple sclerosis, congenital muscular dystrophy, congenital anomalies integument, leprosy, liver cirrhosis with complications, premature infants determined to have medium impairments 3 months after birth, toxic effect of arsenic and its compounds (black foot), motor neurone disease, Creutzfeldt-Jakob disease, and rare diseases [7].

To calculate a Charlson Comorbidity Index (CCI), the first five diagnostic codes (N-Code) of a patient were each multiplied respectively by the scores assigned to 19 different diseases put forth by Charlson et al., and the five resultant figures were then totaled to reach a final index. A higher index was representative of more, or more serious, accompanying diseases [8]. Statistical significance was determined by 2-tailed tests (P<0.05). SPSS 18.0 software was used to conduct the statistical analyses.

## Results

### Demographics

Of the 3,986 patients who were hospitalized because of acute poisoning by insecticides (E863.1) (2,886/3,986) and herbicides (E863.5) (1,100/3,986) between 1999 and 2008 in Taiwan, there were 168 pesticide poisoning deaths during hospitalization.

Table 1 provides demographic information. Males, the dominant group, accounted for 75.4% of pesticide poisoning hospitalizations. The overall mortality rate was higher for males than females.

**Table 1:** Demographics of the study subjects, 1999–2008.

	Insecticides (E863.1)						Herbicides (E863.5)						Total					
	(n = 2,886; 15,884 person-days)						(n = 1,100; 5,699 person-days)						(n = 3,986; 21,583 person-days)					
	N	%	Mean ± SD	No. of deaths	Mortality rate per 1,000 person-days	Fatality (%)	N	%	Mean ± SD	No. of deaths	Mortality rate per 1,000 person-days	Fatality (%)	N	%	Mean ± SD	No. of deaths	Mortality rate per 1,000 person-days	Fatality (%)
Sex																		
Male	2185	75.71		45	3.82	2.06	821	74.64		84	19.52	10.23	3,006	75.41		129	8.02	4.29
Female	701	24.29		19	4.62	2.71	279	25.36		20	14.34	7.17	980	24.59		39	7.09	3.98
Age group			54.69±16.48						49.34±19.98						53.22±17.07			
0–4	29	1.00		0	0	0	17	1.55		0	0	0	46	1.15		0	0	0
5–14	12	0.42		0	0	0	5	0.45		0	0	0	17	0.43		0	0	0
15–24	90	3.12		2	3.84	2.22	56	5.09		8	26.49	14.29	146	3.66		10	12.15	6.85
25–44	659	22.83		15	4.46	2.28	389	35.36		43	19.57	11.05	1,048	26.29		58	10.44	5.53
45–64	1218	42.20		17	2.75	1.40	383	34.82		28	16.15	7.31	1,601	40.17		45	5.68	2.81
65+	878	30.42		30	5.27	3.42	250	22.73		25	17.72	10.00	1,128	28.30		55	7.75	4.88
Charlson Comorbidity Index, CCI			0.29±0.79	64	4.03	2.22			0.33±0.90	104	18.25	9.45			0.31±0.83	168	7.78	4.23
0	2,309	80.01		43	3.72	1.86	888	80.73		83	18.90	9.35	3,197	80.21		126	7.90	3.94
1	427	14.80		12	3.92	2.81	136	12.36		14	16.70	10.29	563	14.12		26	6.67	4.62
2	84	2.91		5	7.85	5.95	37	3.36		4	16.74	10.81	121	3.04		9	10.27	7.44
3+	66	2.29		4	6.30	6.06	39	3.55		3	13.04	7.69	105	2.63		7	8.09	6.67
Living area																		
Northern Taiwan	266	9.22		13	6.70	4.89	117	10.64		15	18.36	12.82	383	9.61		28	10.16	7.31
Central Taiwan	1839	63.72		32	3.23	1.74	698	63.45		68	20.51	9.74	2,537	63.65		100	7.56	3.94
Southern Taiwan	632	21.90		13	4.04	2.06	155	14.09		14	15.68	9.03	787	19.74		27	6.56	3.43
Eastern Taiwan	144	4.99		6	7.54	4.17	129	11.73		7	10.45	5.43	273	6.85		13	8.87	4.76
Offshore Islands	5	0.17		0	0	0	1	0.09		0	0	0		0.15		0	0	0
Level of care																		
Medical center	602	20.86		15	3.31	2.49	434	39.45		56	20.79	12.90	1,036	25.99		71	9.83	6.85
Regional hospital	1176	40.75		34	4.72	2.89	438	39.82		42	20.52	9.59	1,614	40.49		76	8.21	4.71
Local hospital	1108	38.39		15	3.61	1.35	228	20.73		6	6.26	2.63	1,336	33.52		21	4.11	1.57
Surgical treatment																		
Yes	787	27.27		49	6.28	6.23	409	37.18		69	24.71	16.87	1,196	30.01		118	11.14	9.87
No	2099	72.73		15	1.85	0.71	691	62.82		35	12.04	5.07	2,790	69.99		50	4.55	1.79
Mechanical ventilation																		
Yes	286	9.91		35	10.21	12.24	85	7.73		22	37.80	25.88	371	9.31		57	14.21	15.36
No	2600	90.09		29	2.33	1.12	1015	92.27		82	16.03	8.08	3,615	90.69		111	6.32	3.07
Hemodialysis																		
Yes	24	0.83		3	10.42	12.50	104	9.45		26	47.62	25.00	128	3.21		29	34.77	22.66
No	2,862	99.17		61	3.91	2.13	996	90.55		78	15.14	7.83	3,858	96.79		139	6.70	3.60
Length of hospital stay (Mean ± SD)			5.50±8.50	Death: 4.30±6.72					5.18±6.71	Death: 5.58±8.27					5.41±8.04	Death: 5.09±7.72		

With respect to age, individuals 15 to 24 years old who experienced herbicide poisoning had a higher mortality rate than other age groups. The mortality rate for herbicide poisoning was consistently higher than that for insecticide poisoning. The proportion of patients hospitalized in Central Taiwan was higher than that of other areas. For patients hospitalized in medical centers or regional hospitals, the mortality rate was higher than that of local hospitals. Of the patients, 9.91% (286/2,886) and 7.73% (85/1,100) were treated with mechanical ventilation among insecticide and herbicide poisonings, respectively. There was a higher proportion of herbicide poisoning patients (9.45%) who underwent hemodialysis treatment compared with insecticide poisoning patients (0.83%). The average hospital stays were 5.5 days and 5.2 days for the insecticide and herbicide poisoning groups, respectively.

### All-cause and cause-specific mortality rates

The all-cause and cause-specific mortality rates for the entire inpatient cohort separated into the two poisoning groups are presented in Table 2. There were 168 deaths over 21,583 person-days of follow-up for an overall death rate, corresponding to 7.8 deaths per 1000 person-days. The all-cause mortality rate for the herbicide poisoning patients was higher than that of insecticide patients. The top two causes of mortality for insecticide poisoning patients were toxic effects of the organophosphates (3.5 per 1000 person-days) and coma (0.1per 1000 person-days); the major causes of mortality for herbicide poisoning patients were the toxic effects of other pesticides (16.3 per 1000 person-days) and the toxic effects of organophosphates (1.4 per 1000 person-days). There were 89% (93 out of 104) of those who died after herbicide poisoning, the cause of death is stated as ‘toxic effects of other pesticides, not elsewhere classified’ that deaths due to herbicides in general (and including paraquat and glyphosate) are classified in this category.

**Table 2:** All-Cause and Cause-Specific Mortality (n = 3,986).

Cause of death	Insecticides		Herbicides	
	(E863.1) (n = 2,886; 15,884 person-days)		(E863.5) (n = 1,100; 5,699 person-days)	
	No. of deaths	Mortality rate (per 1,000 person-days)	No. of deaths	Mortality rate (per 1,000 person-days)
All Causes	64	4.03	104	18.25
Toxic effect of organophosphates (989.3)	56	3.53	8	1.40
Toxic effect of other pesticides, not elsewhere classified (989.4)	0	0	93	16.32
Anoxic brain damage (348.1)	1	0.06	0	0
Cardiac arrest (427.5)	1	0.06	0	0
Congestive heart failure (428.0)	0	0	1	0.18
Pneumonitis due to inhalation of food or vomiting (507.0)	1	0.06	0	0
Acute respiratory failure (518.81)	1	0.06	0	0
Alcoholic liver damage, unspecified (571.3)	0	0	1	0.18
Acute renal failure (584.9)	1	0.06	0	0
Coma (780.01)	2	0.13	0	0
Toxic effect of unspecified lead compound (984.9)	1	0.06	1	0.18

### Prognostic factors of mortality in pesticide poisoning patients

**Figure 1:**
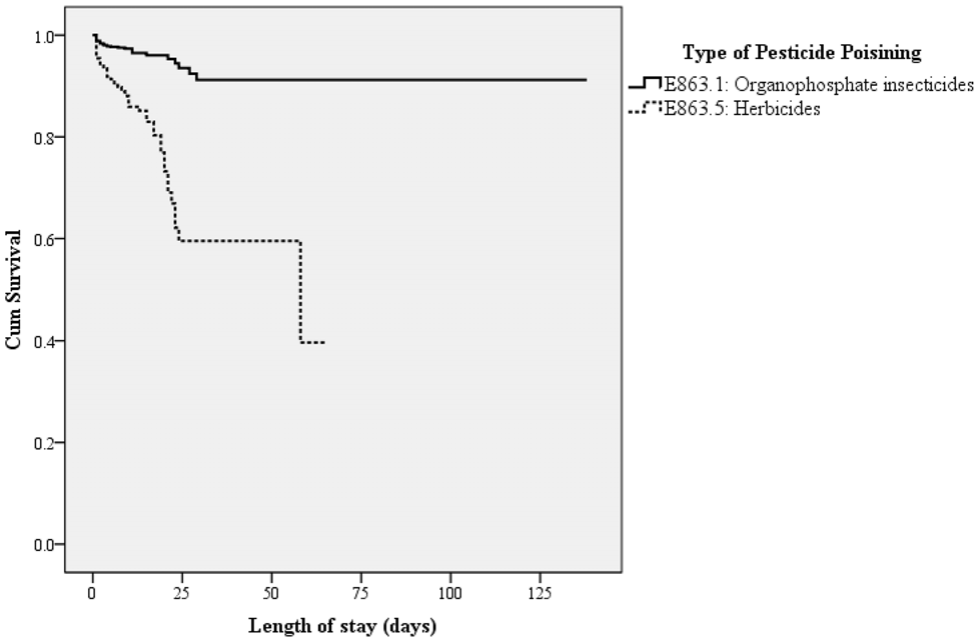
Kaplan-Meier analysis of overall survival for 3,986 inpatients divided into organophosphate insecticides and herbicides poisoning groups (log-rank *p*<0.001).

As shown in Figure 1, the overall survival for herbicide inpatients was significantly worse than that for insecticide poisoning patients (p<0.001). The median survival time of length of stay for herbicide was 60 days. Table 3 shows the Cox regression hazard model evaluating cause of poisoning, sex, age, CCI, living area, level of care, mechanical ventilation treatment and their association with mortality risk.

**Table 3:** Predictors of mortality for pesticide inpatients in Taiwan, 1999–2008^a^.

Variables^b^	B	Adjusted HR	95% CI	p-Value
Type of pesticide poisonings				
E863.1: insecticides		Reference^c^		
E863.5: herbicides	1.52	4.58	3.29–6.37	<0.001
Sex				
Male	0.07	1.08	0.75–1.54	0.70
Female		Reference		
Age group				
0–4		NA		
5–14		NA		
15–24	0.42	1.53	0.76–3.08	0.24
25–44	0.10	1.11	0.75–1.64	0.61
45–64	−0.35	0.70	0.47–1.05	0.09
65+		Reference		
Charlson Comorbidity Index (CCI)				
0		Reference		
1	0.04	1.04	0.67–1.59	0.87
2	0.28	1.33	0.66–2.67	0.43
3+	0.05	1.05	0.48–2.29	0.90
Living area				
Northern Taiwan		Reference		
Central Taiwan	−0.33	0.72	0.47–1.10	0.13
Southern Taiwan	−0.30	0.74	0.43–1.27	0.27
Eastern Taiwan	−0.22	0.80	0.41–1.58	0.52
Offshore islands		NA^d^		
Level of care				
Medical center	0.50	1.65	1.00–2.75	0.04
Regional hospital	0.58	1.79	1.09–2.91	0.02
Local hospital		Reference		
Mechanical Ventilation				
Yes	1.35	3.85	2.73–5.42	<0.001
No		Reference		

a Using Cox proportional hazards models.

b Type of pesticide poisoning (insecticides/herbicides), age (0–4, 5–14,15–24, 25–44, 45–64, 65 or older), sex (male/female), Charlson Comorbidity Index (CCI), living area (northern, central, southern, eastern, offshore), level of care (medical center, regional hospital, local hospital), mechanical ventilation treatment (yes/no).

c Reference group.

d NA: no mortality cases.

Overall, the significant prognostic factors of all-cause mortality in this population were herbicide poisoning (HR = 4.58, 95% CI 3.29 to 6.37), and receiving mechanical ventilation treatment (HR = 3.85, 95% CI 2.93 to 6.00). With regard to the level of medical care, patients hospitalized in medical centers or regional hospitals had hazards ratios of 1.65 (95% CI 1.00–2.75) times and 1.79 (95% CI 1.09–2.91) times compared with patients hospitalized in local hospitals.

## Discussion

This was a nationwide population-based study, allowing us to trace the medical services received by all inpatients after unintentional poisoning. The cumulative survival rate for herbicide inpatients was significantly worse than that of insecticide poisoning patients. The mortality rate for herbicide exposure was almost fivefold higher than that of insecticide exposure. The factors associated with inpatient mortality were herbicide poisonings, level of medical care, receiving ventilation and hemodialysis treatment.

### Cause-specific mortality and prognostic factors

The high mortality rate for herbicide poisoning was in concordance with previous reports [3], [9]. One study that recruited various poisoning patients through emergency departments in Southern Taiwan showed that herbicides caused the highest fatality rate [9]. Paraquat and glyphosate were found to be the most frequent herbicides involved in fatalities.

Hemodialysis is one among the treatments for paraquat poisoning [10], as a consequence, much more patients suffered from severe herbicide poisoning in this study received hemodialysis therapy. It has been shown that patients exposed to glyphosate through oral ingestion died more frequently than patients exposed via other pathways. Shock and respiratory failure accounted for most fatalities [4]. Intubation and mechanical ventilation are essential treatment for the most severe poisoning cases [11]. Clinical features such as the development of respiratory distress, impaired consciousness, pulmonary edema, shock, arrhythmias, renal failure requiring hemodialysis and the presence of infiltrate on chest x-ray were some parameters associated with poisoning-related fatality for glyphosate poisoning patients [11]. In addition, hemodialysis and hemoperfusion are two elimination methods used to treat paraquat poisoning patients, but these treatments are unlikely to change the clinical course. The case-fatality rate is quite high despite different variations in treatment [10]. Our finding is consistent with above reports that hemodialysis treatment is associated with a high herbicide poisoning fatality rate. Death among organophosphate poisoning patients is chiefly related to respiratory failure, central nervous system depression, seizures or ventricular arrhythmias. The pathogenesis of respiratory distress and failure was related to excessive bronchial secretions, bronchospasm, pulmonary edema, aspiration pneumonia and paralysis of respiratory muscles. This association might explain that ventilation treatment is another indicator of serious complications of organophosphate and herbicide poisonings.

With regard to the level of medical care, patients hospitalized in medical centers or regional hospitals had hazards ratios 1.65 times or 1.79 times compared with patients hospitalized in local hospitals. We found that only 29% and 50% of patients were transferred to a higher level of medical service among insecticide and herbicide poisonings, respectively. The primary hospital might have lacked of medication, equipment, or staff [12]. The level of medical care used in our study represented only the most recent hospitalization. Our results could be influenced by the fact that higher medical care facilities are prone to an over-representation of severe poisoning cases. For poisoning by highly toxic pesticides such as paraquat, patients transferred to a higher level of medical service might not experience better outcomes.

It is critically important to know exactly the type of chemical involved in a pesticide poisoning. However, further information on the specific type of pesticide within the categorization of organophosphate or herbicide was not available. Currently, organophosphate insecticides are the most common type of insecticides in Taiwan including Disulfoton,Terbufos, Azinphos-Methyl, Mevinphos, Methidathion, Demeton-S-Methyl, Methamidophos, Phorate, Dicrotophos, Trichlorfon. There were categorized on the basis of the WHO pesticide hazard classification [13] as moderately hazardous to extremely hazardous classes. Paraquat and glyphosphate are two types of herbicides that are used most frequently in Taiwan [14].

### Intentional vs. non-intentional

Lin et al. (2008) reported that adults experienced more intentional OP exposures (67%) and that ingestion was the most common route of exposure (74.5%) based on the network of Taiwan's Poison Control Centers (PCC). Lee et al. (2008) reported a similar proportion (66.1%) of poisoning exposures involving suicidal intent. However, our study included only pesticide poisoning hospitalizations in which the poisoning was non-intentional.

There was a large variation in the case fatality rate among pesticide poisonings, ranging from 0% to 42%. Even for the same chemical and/or World Health Organization (WHO) toxicity classification of pesticides and for those used for similar agricultural indications, a marked variation in lethality was observed [15]. Patients can come into contact with toxic substances intentionally or by accident. Unlike the situation in which a substance is deliberately administered, in accidental exposure cases, there is usually only a poor understanding of the pesticides to which a subject is exposed, both in a qualitative and a quantitative sense.

In addition, the occurrence and magnitude of harmful effects and the part of the patients' bodies in which these effects arise depend on the form in which the patients are exposed to the pesticides. The form of exposure is determined by the environmental compartment in which the victim exists, the physical state of the pesticide (gas or liquid), the physicochemical prosperities of the pesticide (soluble in water or not) and the nature of contact (single or prolonged). Furthermore, whether a pesticide is absorbed, and at what rate, is determined by the barriers that are encountered along the route of entry into the body.

For 8 persons categorized as herbicide poisoning, the cause of death was indicated as ‘toxic effects of organophosphates for 93 (out of the 104 deaths) as ‘toxic effects of other pesticides, not elsewhere classified’. This could be explained either by misclassification of the type of poisoning and/or cause of death as well as by the presence of two types of substances, a herbicide and organophosphate. It has been previously reported that glyphosate herbicide poisoning patients received atropine or pralidoxime therapy [4], [16] even though atropine and pralidoxime are not antidotes for glyphosate poisoning. These reports imply that some physicians confuse glyphosates with organophosphates.

### Limitations

Several limitations need to be considered in the interpretation of our findings. First, no further information on the specific type of pesticide within the categorization of organophosphate or herbicide was available, and no information was available about the exposure route and the amount of exposure. Second, hospitalization data have an over-representation of severe poisonings and severe symptoms because of self-selection to the hospital policlinics as well as admission to the hospital care. The quality of diagnostic practice and criteria for hospitalization may vary and it is possible that pesticide poisoning is under-reported in Taiwan.

Third, the comorbidity diagnoses, which rely completely on the claims data reported by the physicians or hospitals, may be less accurate than if all individuals had been assessed using a single standardized procedure [17]. Health professionals may not receive much training in environmental toxicology or pesticide poisoning. The signs and symptoms of pesticide poisoning often resemble those of more common conditions, which may be diagnosed preferentially. The NHIRD used discharge diagnoses provided by treating physicians; no standardized criteria were used to define hospitalization cases. This lack of standardization increases the probability for case misclassification. The NHIRD, designed as an administrative dataset, does not include some important individual characteristics for further analyses such as smoking and alcohol consumption, all of which may contribute to death or hospitalization. The mortality and hospitalization rates may be underestimates because of the possible under-reporting of cases in the databases used in the analyses. Administrative databases are known to be subject to possible undercoding and overcoding errors [18]. The difficulty of receiving reimbursement through workers' compensation may also bias health care providers' diagnoses and the reporting of episodes of unintentional pesticide poisoning. The health care professionals may fear that their patients may be subject to retaliation.

### Strengths of this study

Litchfield [19] categorized studies on acute pesticide poisoning in agriculture into three categories: clinical case reports, descriptive epidemiology studies, and cross-sectional studies. Several studies conducted in China, India, and Taiwan have been centered on hospital-based case reports [1], [3], [9], . These reports included insufficient information on the source population to estimate the poisoning rates. A particular strength of the current study is the use of a nationwide population-based dataset, allowing us to trace the medical services received by all patients after poisoning. Large computerized databases derived from this system by the Bureau of National Health Insurance, Taiwan (BNHI) and maintained by the National Health Research Institutes, Taiwan, are provided to scientists in Taiwan for research purposes.

NHIB established a uniform system to control the quality of medical services and coding. Chou et al. (2003) reported the accuracy of the comorbidity in the claims data using medical records as the gold standard [22]. The kappa values for all of the demographic data between two abstractors were greater than 95%. In addition, the kappa values for 18 comorbidities were more than 84% in inter-abstractor reliability. The agreement of kappa value of comorbidities between medical records and claims data were in a range between 0.31 and 0.86. Authors concluded the validity of comorbidity data in NIH claims data was in the range of moderate and substantial agreement. Several studies have been published also supported the strong validity of the NHIRD [23], [24].Using the same ICD-9-CM codes over the study period maintained the internal validity of the temporal trend analyses. Except for the limited information of the route of exposure of pesticide poisoning, the present study is the most complete nationwide population-based study conducted to assess the risk of inpatient mortality and to identify some prognostic factors of inpatient mortality among insecticide and herbicide pesticide poisonings.

### Conclusions

The major causes of mortality for insecticide poisonings were the toxic effect of organophosphates and coma, whereas the major causes of mortality for herbicide poisonings were the toxic effect of other pesticides and the toxic effect of organophosphates. The factors associated with inpatient mortality were herbicide poisoning and receiving further medical treatment; however, we could not further classify the different substances in the organophosphate insecticide and herbicide categories. We demonstrated that herbicides stand out as the dominant agent for poisoning-related fatalities. From public health perspective, there is an obvious need to strengthen the efforts to get more information on the details of herbicides for this database. This would allow identification of the most hazardous herbicides and could lead to better treatment and prevention of accidents in the future. Furthermore, the control of and limiting access to herbicide agents and developing appropriate therapeutic regimens, including emergency care, should be priorities. Physicians and general practitioners should be aware and capable of recognizing these types of poisonings early because such poisonings are rapidly fatal unless correctly diagnosed and treated. More research on chronic pesticide exposure is also needed because there is still relatively little known about the health effects of these types of poisonings.
